# Exploitive Adolescent Labor and Labor Trafficking: Clinical Perspectives for Adolescent Healthcare Providers

**DOI:** 10.1016/j.jadohealth.2025.12.264

**Published:** 2026-02-05

**Authors:** Romina Barral, Michele A. Kelley, Abigail English, Marissa Raymond-Flesch, Tonya Chaffee, Veronica Svetaz, Tamera Coyne-Beasley, Nuray Kanbur

**Affiliations:** a Division of Adolescent and Young Adult Medicine, Children’s Mercy Hospital and Clinics, University of Missouri-Kansas City School of Medicine, Kansas City, Missouri; b School of Medicine, University of Kansas Medical Center, Kansas City, Kansas; c School of Public Health, University of Illinois Chicago, Chicago, Illinois; d Department of Maternal and Child Health, Center for Adolescent Health and the Law, University of North Carolina at Chapel Hill Gillings School of Global Public Health, Chapel Hill, North Carolina; e Division of Adolescent and Young Adult Medicine, Philip R. Lee Institute for Health Policy Studies, University of California, San Francisco, California; f Department of Pediatrics, Teen and Young Adult Health Center, CASARC, Zuckerberg San Francisco General, University of California, San Francisco, California; g Department of Family Medicine and Community Health, University of Minnesota, Minneapolis, Minnesota; h Division of Adolescent Medicine, University of Alabama at Birmingham, Birmingham, Alabama; i Division of Adolescent Health, Department of Pediatrics, University of Ottawa, Child and Adolescent Eating Disorders Program, Children’s Hospital of Eastern Ontario (CHEO), Ottawa, Ontario, Canada

Health-care providers are aware that many adolescents work, including in legitimate jobs for monetary compensation or to help their families in a variety of healthy ways. Literature suggests that working less than 20 hours a week can be developmentally appropriate and helpful for midadolescents to late adolescents [1]. Important questions remain: When does work become problematic? How do we screen for problematic work in adolescents, like exploitive or trafficked work conditions?

There is overlap between exploitive adolescent labor, understood broadly, and adolescent labor trafficking; this commentary addresses clinical aspects of both. This commentary reviews basic definitions and trends and offers guidance about common red flags for exploitive adolescent labor as well as possible resources and strategies for helping youth and families in these situations.

## Definitions

A major challenge in understanding and addressing exploitive adolescent labor is establishing shared definitions and regular data collection across and within countries. In addition, there is an intersection among child labor, child labor trafficking, and child sex trafficking that contributes to confusion [2]. Child sex trafficking is a form of child exploitation, about which much has been written on the critical role of health-care providers [3]. However, this clinical commentary is focused explicitly on the underaddressed issues of exploitive adolescent labor and labor trafficking. Although many official definitions use the term “child,” this commentary specifically addresses concerns related to the adolescent age group, which represents a subset of children.

Basic definitions in international and United States (US) law highlight why clinicians need to be aware of the potential impact of exploitive labor on their adolescent patients. The 1989 United Nations Convention on the Rights of the Child articulated “the right of the child to be protected from economic exploitation and from performing any work that is likely to be hazardous or to interfere with the child’s education, or to be harmful to the child’s health or physical, mental, spiritual, moral or social development.” [4] The International Labour Organization International Programme on the Elimination of Child Labour and Forced Labour elaborated on that right in its definition:

“The term “child labour” is often defined as work that deprives children of their childhood, their potential and their dignity, and that is harmful to physical and mental development. It refers to work that: is mentally, physically, socially or morally dangerous and harmful to children; and/or interferes with their schooling by: depriving them of the opportunity to attend school; obliging them to leave school prematurely, or requiring them to attempt to combine school attendance with excessively long and heavy work” [5].

Defining and quantifying exploitive adolescent labor is complicated by overlapping definitions in international and US law and by intersection in the use of terms such as child labor, forced labor, and trafficking. An additional challenge arises with the difficulty of proving the elements—such as “force, fraud, or coercion”—that are needed to establish human trafficking [6].

## Global Trends

According to recent estimates, exploitive adolescent labor impacts 59 million adolescents aged 12–17 years [7]. The majority of them (81%, 43.6 million) perform hazardous work that is likely to harm their health, safety, or morals [7]. Overall, boys are a majority of those affected; however, when factoring in household chores, more girls seem to be affected by exploitive adolescent labor [7]. Although exploitive adolescent labor is a long-standing problem, the exploitation of minors for labor both in the US and internationally is growing as a source for an inexpensive workforce in the setting of economic disparities and increased labor shortages. Internationally, a 2024 report noted that all regions, including Asia and the Pacific, Latin America and the Caribbean, and sub-Saharan Africa had seen decreases in exploitive adolescent labor numbers since 2024, though South Saharan Africa continues to lead the numbers for exploitive adolescent labor [7]. Meeting the United Nations Sustainable Development Goal 8.7 target of eliminating child labor by 2030 would require a decline 11 times faster than the one that has occurred since 2020 [7]. In the US, children and adolescents employed in violation of current laws have increased 283% since 2015 [2].

## Causes and Health Consequences of Child Labor and Child Labor Trafficking

A main driver of exploitive adolescent labor is poverty. Exploitive adolescent labor is often seen in industries where cheaper labor is required and immigrants of all ages are exploited, such as in agriculture, construction, domestic work, food packaging, and other dangerous work environments [8]. Although higher rates of exploitive adolescent labor are seen in lower-income countries, wealthier countries, including the US, sustain high rates of exploitive adolescent labor in agriculture and other industries [9]. Adolescents living in poverty and migrant/refugee adolescents are at a higher risk for being forced into exploitive labor or being trafficked, placing them at risk of suffering violence, abuse, and other human rights violations, including sexual exploitation (mainly for girls) and armed forces exploitation (mainly for boys) [10].

Exploitive adolescent labor and labor trafficking often leads to extreme physical and mental harm, and even death. Its toxic stress can have health impacts across the lifespan, including deprivation of fundamental rights: disruption of education, physical injuries, interruption of pubertal and neurocognitive development, and severe impacts on mental health [3]. The health consequences of exploitive adolescent labor may be observed by clinicians without being identified as related to harmful child labor or labor trafficking. Clinicians may not be aware that an adolescent is working and typically are not assessing whether an adolescent’s employment is forced, coerced, or unsafe [11].

## Screening and Interventions

Clinicians are more familiar with and often skilled in assessing child abuse and, in some practices, they also have skills in assessing risk or indicators of sex trafficking. These skills can provide a foundation for evaluating the health and psychosocial risks and impacts of exploitive adolescent labor and trafficking for an individual patient. However, a barrier to screening for many complex psychosocial and socioeconomic factors is that clinicians are often unsure how to respond to a positive screen. Asking about work with implications around illegality should involve a very careful approach, since it can provoke emotions of fear or reactions to being stereotyped or fear of being turned over to “authorities”. This approach is difficult but important and takes trust and means exploring a patient’s involvement in both paid and unpaid work.

Tables 1 and 2 provide guidance about common red flags for exploitive adolescent labor, as well as possible resources and strategies for helping youth and families. Clinicians must understand that adolescents are most likely to be at risk of exploitive labor when families lack appropriate resources for basic survival.

For adolescents living with their families, interventions will most often focus on connecting the patient and family to resources such as food, clothing, shelter, transportation, and education. The response requires structural competency, where a symptom or behavior is seen as determined by structural factors like poverty and scarcity. These resources need to be shared within a collaborative, trauma-informed, rights-based, and culturally appropriate approach to care, with an awareness that most families facing scarcity are more likely to be families that have been historically marginalized, given their increased exposure to negative social determinants of health determined by structural racism and personally mediated racism (with more negative interactions with a health system that amplifies those social disparities by provision of unequal care). A culturally responsive and race-conscious wrap-around approach requires awareness that these families are using a survival mechanism and need a support system.

Similarly, and given the growing trauma-informed collaborative community responses that are addressing adolescent sex trafficking, it would be imperative to include and/or add such responses for those minors who are being exploited and trafficked for labor rather than creating whole new collaborative responses.

For adolescents not living with their families, the response should involve considering the steps as outlined in Table 3.

## Conclusions

Health professionals have a key role in both identifying and responding to exploitive adolescent labor. Increased training and education, as well as the development of health system protocols for screening and linking patients to trauma-informed, collaborative, patient-centered responses are needed nationally and internationally to protect the healthy development of youth from this often-ignored driver of health.

## Figures and Tables

**Table 1:** Risk factors and concerning signs and symptoms (adapted from a systematic literature review of the impacts of child labor on child’s health in low- and middle-income countries [12])

Risk factors for exploitive child labor
● Poverty
● Migrant and refugee children
● Not speaking local language
● Known work in high-risk industries: agriculture, slaughterhouses, mining, construction, warehouses.
● Food insecurity
● Unaccompanied minor/lack of guardianship
Concerning signs/symptoms
● Accompanying adult preventing independent discussion between provider and child/adolescent
● Sleep disturbances, chronic fatigue, poor historian, or unable to tell date/ time/location due to sleep deprivation
● Work interfering with school attendance
● Musculo-skeletal pain/symptoms, wasting, malnutrition, presence of various diseases (anemia, vitamin deficiencies, repeated infections)
● Physical injuries, especially work-related injuries, e.g, overuse/arthritis esp. in young age
● Clinical visits for hazardous exposures, burns, acute pesticide poisoning
● Delayed care or lack of medical care for chronic diseases, overall concerns for abuse
● Worsening asthma, fever/cough, and respiratory morbidities (tuberculosis, hilar gland enlargement/calcification)
● Emotional and behavioral escalations, mood, and anxiety disorders
● HIV/HBV, HCV, other STIs, especially if multiple infections
● Substance misuse (smoking, drug use, high caffeine intake)

HBV = Hepatitis B virus; HCV = Hepatitis C virus; STIs = sexual trasnmitted infections.

**Table 2:** Recommend trauma informed, rights-based, and culturally appropriate universal screening, to determine risk, not disclosure, using some of these screening tools [13,14]

Questions adapted from American Academy of Pediatrics Screening Tools for Child Trafficking and Exploitation:
● I will now ask about work. Many young people have jobs, side hustles, and other things they do, to help bring in money. This work can sometimes affect health or learning or having time for oneself and friends. “The words, ‘work’ and ‘job’ can mean lots of things, from formal jobs in areas like construction or restaurant work, to informal arrangements to clean someone’s home, take care of children, or to help a relative with their job. Sometimes, ‘work’ involves some other activity that a person does for someone else. Have you ever had a job or done work for someone else?”
● “With an affirmative answer, the clinician might continue with: Can you tell me something about it? What did your work involve? Did you have specific work hours? Did you wear any safety equipment (if appropriate)?” Did you get paid for the work you did?
● “Then, move on to more specific questions about possible exploitation: Have you ever had a job, only to find that the work you are doing, or the conditions of your work are not what you were promised? Has anyone associated with your work ever made you feel scared?”
Interviewing and Assessment Strategies:
● “Strategies for Interviewing the Patient Alone” and “Strategies for Working with Minor Patients” are components of HEAL Trafficking’s Protocol Toolkit for Developing A Response to Human Trafficking Victims in Health Care Settings [15].

**Table 3:** Possible interventions — from American Academy of Pediatrics [16]

● What to do with for children and adolescents who are at risk for labor or sex exploitation or trafficking
• Offer anticipatory guidance regarding basic labor rights
• Right to be free from unlawful discrimination and harassment
• Right to a safe workplace free of dangerous conditions, toxic substances, and other potential safety hazards
• Right to be free from retaliation for filing a claim or complaint against an employer, often called "whistleblower” rights.
• Refer to programs for General Educational Development (GED) and college placement, job skills training, and professional attire donation.
• Offer high-risk patients and families community and national resources to address vulnerabilities: https://www.usa.gov/benefits
• If trafficking concerns, consider discussing common trafficking recruitment scenarios, healthy relationships
● What to do if you are suspecting labor and/or sex trafficking case
• Provide support and referral of those at risk or involved in exploitation
• Know local responses and agencies that patients can be referred to and ensure they are trauma informed.
• Refer to state child abuse reporting law or contact local child protective services (CPS) agency for guidance. Mandatory reporting for suspected child labor trafficking is not present in the reporting laws of most US states. Consider risk/benefits here.
• Contact national organizations before reporting for guidance and reporting recommendations:
• National Human Trafficking Resource Center Hotline (1–888-3737–888 or text 233,733)
• Polaris Project (www.polarisproject.org) (sponsors the hotline above)
Shared Hope International (sharedhope.org)
• National Center for Missing and Exploited Youth (www.Missingkids.com)
• Other helpful sources of guidance include
• Staff from state law enforcement task forces on child trafficking
• State or local law enforcement
• CPS agencies
• Local child advocacy centers (see above)
● What to do if your teen is an Undocumented immigrant
• Connect with resources (like https://unidosus.org/ or https://www.americanimmigrationcouncil.org/) that will help them to assess the programs that they might be eligible for, and the risks associated with applying for those programs.
• Link family to legal services
Pro bono attorneys
• Government legal aid
• Nongovernmental organizations: Kids in Need of Defense
• Immigration relief clinics provide medical and mental health forensic evaluations
• Society of Asylum Medicine, https://asylummedicine.com/
• Physicians for Human Rights https://phr.org/
• Refer to organizations that provide immediate relief and aid, protection, and ongoing advocacy for immigrants and refugees
• The International Committee of the Red Cross: https://www.icrc.org/en
• The International Rescue Committee: https://www.rescue.org
